# Dual color optogenetic control of neural populations using low-noise, multishank optoelectrodes

**DOI:** 10.1038/s41378-018-0009-2

**Published:** 2018-06-04

**Authors:** Komal Kampasi, Daniel F. English, John Seymour, Eran Stark, Sam McKenzie, Mihály Vöröslakos, György Buzsáki, Kensall D. Wise, Euisik Yoon

**Affiliations:** 1 Department of Biomedical Engineering, University of Michigan, Ann Arbor, MI 48105 USA; 2 Center for Micro and Nanotechnology, Lawrence Livermore National Laboratory, Livermore, CA 94550 USA; 3 NYU Neuroscience Institute, School of Medicine, East River Science Park, Alexandria Center, 450 East 29th St, 9th Floor, New York, NY 10016 USA; 4 Department of Electrical Engineering and Computer Science, University of Michigan, Ann Arbor, MI 48105 USA; 5 Department of Physiology and Pharmacology, Sackler Faculty of Medicine, Tel Aviv University, 69978 Tel Aviv, Israel; 6 Sagol School of Neuroscience, Tel Aviv University, 69978 Tel Aviv, Israel

## Abstract

Optogenetics allows for optical manipulation of neuronal activity and has been increasingly combined with intracellular and extracellular electrophysiological recordings. Genetically-identified classes of neurons are optically manipulated, though the versatility of optogenetics would be increased if independent control of distinct neural populations could be achieved on a sufficient spatial and temporal resolution. We report a scalable multisite optoelectrode design that allows simultaneous optogenetic control of two spatially intermingled neuronal populations in vivo. We describe the design, fabrication, and assembly of low-noise, multisite/multicolor optoelectrodes. Each shank of the four-shank assembly is monolithically integrated with 8 recording sites and a dual-color waveguide mixer with a 7 × 30 μm cross-section, coupled to 405 nm and 635 nm injection laser diodes (ILDs) via gradient-index (GRIN) lenses to meet optical and thermal design requirements. To better understand noise on the recording channels generated during diode-based activation, we developed a lumped-circuit modeling approach for EMI coupling mechanisms and used it to limit artifacts to amplitudes under 100 μV upto an optical output power of 450 μW. We implanted the packaged devices into the CA1 pyramidal layer of awake mice, expressing Channelrhodopsin-2 in pyramidal cells and ChrimsonR in paravalbumin-expressing interneurons, and achieved optical excitation of each cell type using sub-mW illumination. We highlight the potential use of this technology for functional dissection of neural circuits.

## Optogenetics: Compact optoelectrode allows simultaneous control of separate neuron types

A novel compact, multi-site optoelectrode was able to achieve control of different types of neurons in awake, behaving mice. Optogenetics allows the activity of neurons in living tissue to be controlled by means of light, and optoelectrodes are devices used to achieve such control. A team headed by Euisik Yoon at the University of Michigan in the United States developed a novel type of optoelectrode and implanted it into the brains of awake mice. With the low-noise, multi-site optoelectrode, the authors were able to attain simultaneous optogenetic control of two distinct but intermingled types of neurons. The team believes that its technology can be used for neural circuit integration and help understand the organization and function of complex neural circuits, which constitutes an important new area in neuroscience research.

## Introduction

Optical perturbation of genetically-defined neuron types^1,2^ combined with large-scale recordings^3–5^ has become a widespread tool for neural circuit analysis. Optogenetics enables specific light wavelengths to activate microbial opsins. When combined with molecular targeting strategies such as Cre-LoxP systems, the technique can provide cell-type specificity and high temporal resolution^6,7^ that electrical and other stimulation techniques cannot. Novel opsins are available in a wide range of spectral sensitivities^8–14^ allowing precise interrogation of neural circuit computations^5,15^ and deciphering circuit mechanisms^16–18^. Successful multi-opsin experiments require opsins to have sufficient spectral separation and sensitivity to produce robust and independent spiking within the optical power range offered by the optoelectronic toolset. While these constraints increase the design complexity of experiments, they enable in vivo multicolor experiments not previously possible.

Combining precise light delivery with reliable electrophysiological readout is a technological challenge^5,19,20^. Early optoelectrode designs were either bulky and lacked spatial precision for neural optical stimulation^21–23^ and/or incorporated the use of fibers^4^ which limits scaling. To overcome these limitations, optoelectrode technology has made a recent shift to fiber-less approach of optical stimulation, where light sources are integrated directly on neural probes to offer higher-density scalable devices^4,24–27^. While fiber-less approaches offer attractive optoelectrode solutions, they are challenging to implement for implantable devices for several reasons. First, on-probe light sources generate heat and risk thermal damage to the surrounding tissue during operation. Second, the close proximity of electrical traces on a compact scale makes them susceptible to electromagnetic interference (EMI) coupling, generating stimulation-locked artifacts. EMI-induced artifacts may obscure or distort neural activity near the stimulation site for tens or hundreds of milliseconds. Third, packaging multiple sources of different wavelengths (for independent control of different neuron types) in a single micro-assembly poses design challenges of its own. The current state-of-the-art optoelectronic devices offer scalable and high-resolution illumination but are monochromatic^25,28^, limiting their capability to manipulate more than one cell type. We recently reported multicolor optoelectrode approaches for independent control of different neuron types^4,27^ but they have been limited to only one color per site^4^, or to a single site with multiple colors yet with strong stimulus-locked artifacts^27^.

**Fig. 1:**
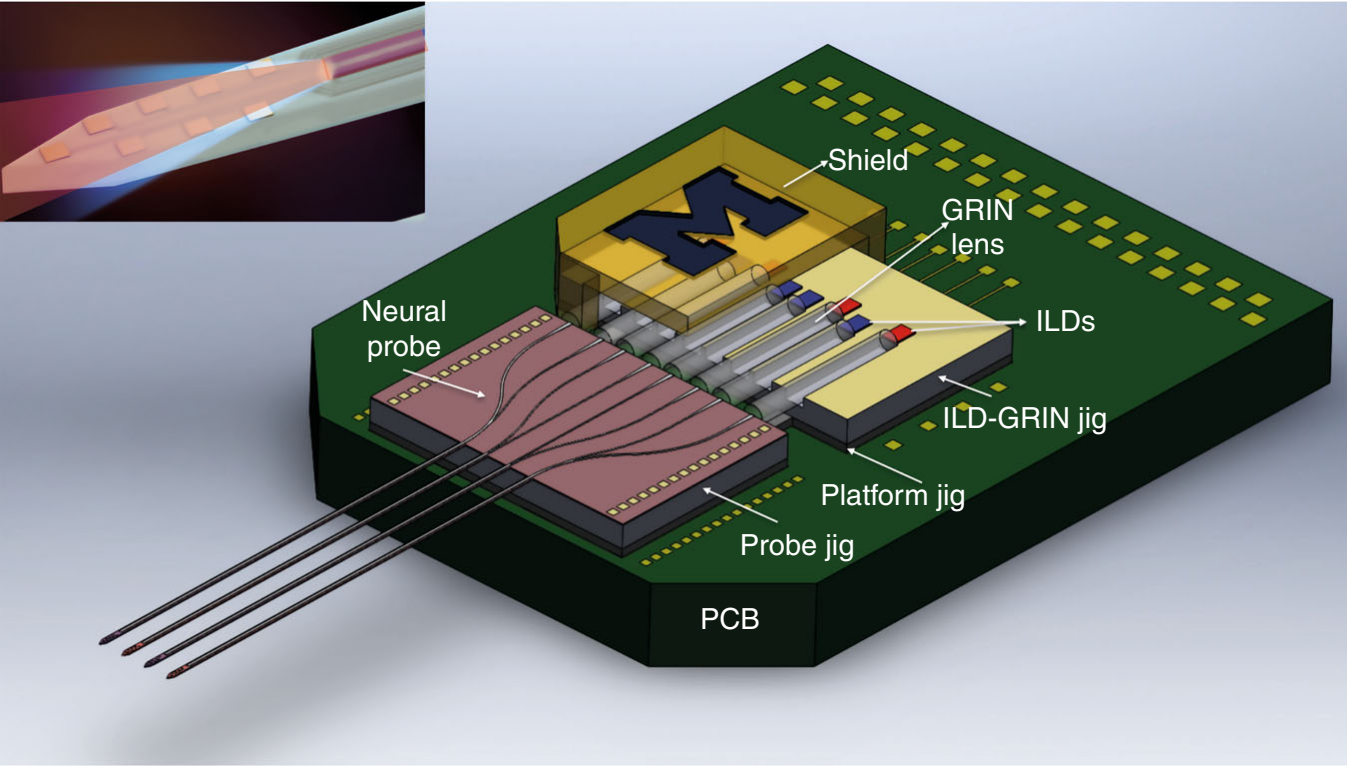
Schematic of multishank multicolor fiberless optoelectrode assembled on a printed circuit board (PCB). The inset shows the magnified probe tip with dual-color light emission at the waveguide tip

**Fig. 2:**
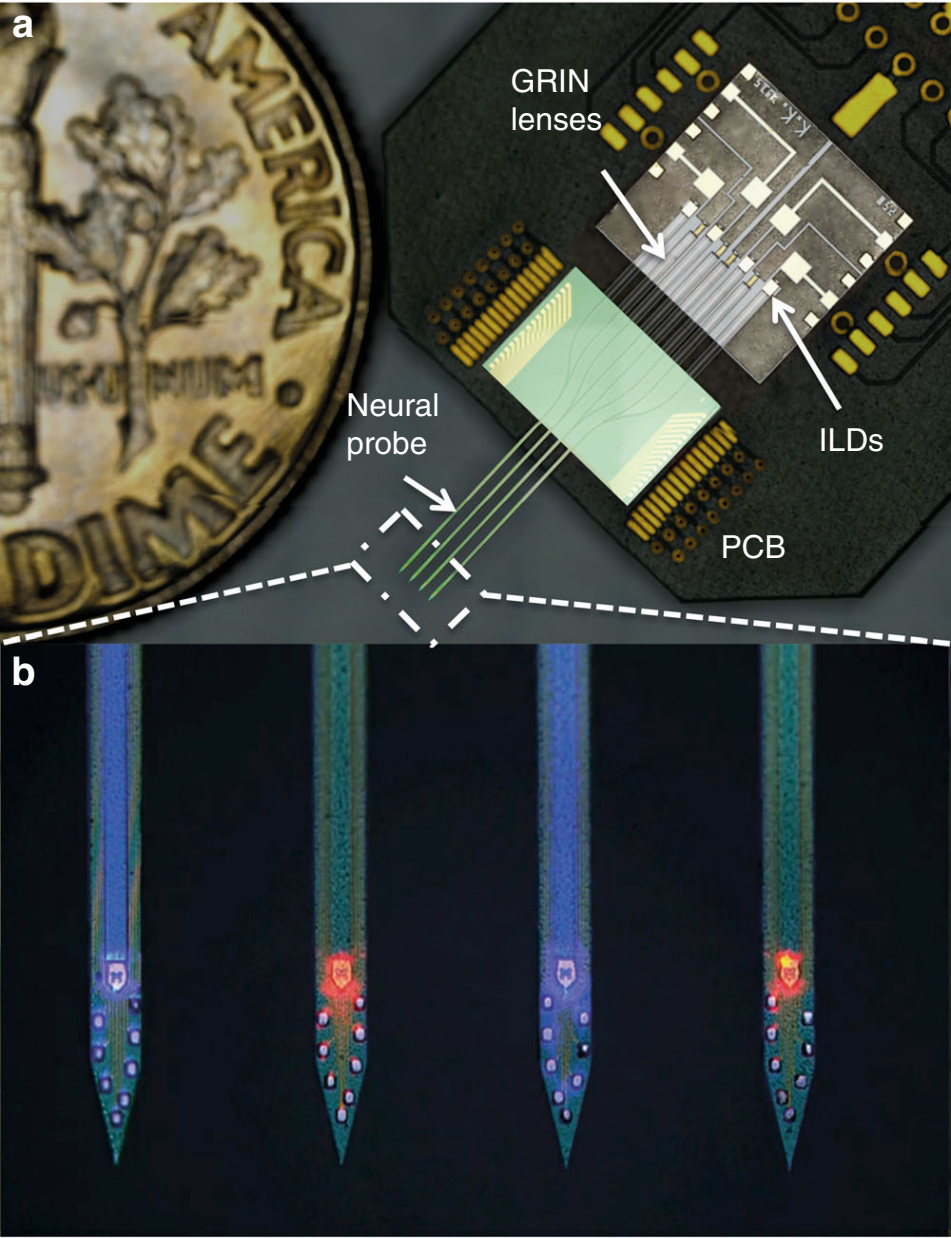
Working device prototype. **a** Assembled device prototype on a PCB, compared to a dime in size. **b** Enlarged view of the probe shank tips with multi color light illuminating from the 30 μm x 7 μm waveguide tips. Also, see Supplementary Movie S1

Here, we present high-density four-shank optoelectrodes with integrated 405 and 635 nm injection laser diode (ILD) light sources for dual color local circuit analysis. As in our previous contribution^27^, the design involves coupling compact ILDs to monolithic dielectric optical mixer waveguides via GRIN lenses. In contrast to the previous single-shank version, the present device has two ILDs for each of four shanks and enables high quality recordings with significantly reduced stimulation artifacts during bench testing (under 100 μV). Importantly for reduced tissue reaction damage, each shank is only 70 μm wide, 22 μm thick, with inter-shank pitch of 300 μm. A compact (5 × 5 mm) “ILD-GRIN jig” houses eight ILD-GRIN pairs. Precise ILD-GRIN alignment was achieved using a die-bonding tool (a.k.a., flipchipper) that aligned ILDs with sub-micrometer accuracy. The assembled ILD-GRIN components and the PCB can also be reused which is another improvement over our past assembly technique. Figure 1 shows the schematic of the GRIN-based optoelectrode array, and Fig. 2 shows the assembled prototype. An optical mixer enables multicolor illumination at a common port (7 × 30 μm) (Supplementary Movie S1), which was used to activate two spatially intermingled cell types in hippocampal CA1 region of awake behaving mice: pyramidal cells (PYR) expressing the blue-light sensitive Channelrhodopsin-2, and parvalbumin-expressing (PV) cells expressing the red-light sensitive opsin ChrimsonR. Our results demonstrate that these novel optoelectrodes can be used for neural circuit interrogation that requires the parametric control of two types of neurons in awake mammals.

## Materials and methods

### Fabrication and assembly

The details of fabrication process are discussed in previous work^27^. Our probe fabrication process allows definition of platinum/iridium electrode sites 2 μm deep in dielectric films, greatly reducing the intensity of waveguide light directly hitting the electrodes and causing recording artifacts due to potential photoelectrochemical effect^29^. The choice of SiON waveguides over polymer waveguides^30,31^ was made as SiON does not absorb light in the UV-blue range^32–34^ and suffers minimal degradation in biological environments.

The assembly of all microfabricated components, within their respective tolerance ranges^27^, was achieved by photo-lithographically defined geometries during microfabrication and precise assembly techniques with the aid of flip-chip bonder and micromanipulators. The ILDs were eutectic bonded (In-Au eutectic at 200 °C) on the released

ILD-GRIN jigs using a flip-chip bonder (Lambda Flipchip bonder, Finetech, Germany) to achieve sub-micron precision and minimum handling damage. The ILD-GRIN jigs with assembled ILDs were annealed in forming gas environment to prevent indium oxidation, hence providing a stronger indium-gold eutectic with improved electrical properties. Following optical assembly of ILDs, GRINs and waveguides, brass shield (Fig. 1) was put in place over the ILD-GRIN jig assembly and grounded to PCB ground. The platform jig and ILD GRIN jig were also electrically connected to the PCB ground using wirebonds and conductive silver epoxy. The assembled devices were wire-bonded on the PCB. Two Omnetics connectors: a 36-pin male for 32 neural recording channels and reference and ground and a 12-pin male for driving and grounding 8 ILDs; (A79022-001 and A79624-001; Omnetics Connector Corporation, Minneapolis, MN, USA) were soldered to the PCB via flexible wires (36744MHW, Phoneix wires Inc, South Hero, VT, USA) for electrical interfacing with an external driver and amplifier.

Our assembly approach is also modular. The assembled ILD-GRIN packages on the PCB can also be reused from device to device to facilitate a cost and labor effective solution. The neural probes are assembled on the PCBs using an acetone dissolving epoxy and PDMS, which can be readily removed/peeled off to replace and re-align a new waveguide neural probe on the same assembly. This probe replacement can be done if the probe accidently breaks during implantation or retrieval and thus the PCB assembly of ILDs and GRIN lenses are all conserved. The reusability of the technique was verified experimentally by assembling more than one probe on the same PCB assembly (with all assembled components) and achieving optical power values at all shanks within ~12% accuracy.

### In vivo recording procedures

All animal procedures were approved by the New York University Animal care and Facilities committee. PV-Cre mice (B6;129P2-Pvalbtm1(cre)Arbr/J; JAX Labs, Maine) were injected in dorsal CA1 (coordinate, in mm from bregma: −1.75 posterior, 2.0 mm left) with two AAVs, encoding Cre dependent ChrimsonR (AAV5-hSyn-FLEX-ChrimsonR-tdTomato) and CaMKII promoter driven ChR2 (AAV5-CaMKIIa-hChR2(H134R)-EYFP), resulting in expression of ChrimsonR in PV + interneurons and ChR2 in pyramidal neurons (Viruses were sourced from the University of North Carolina Vector Core^35^). Animals were additionally implanted with a titanium head plate^36^ and stainless steel ground wire places above the cerebellum. Mice were habituated to head fixation over the course of one week. After habituation, mice were head-fixed, and the electrode was lowered to dorsal CA1. Baseline recording was obtained, after which stimulation with 405 and 635 nm light was made at different intensities and durations. Bench laser drivers (LDC202C from Thorlabs, Inc) and RHD2164 amplifier board with RHD2000 were used for ILD stimulation. Neural data were acquired at 20 kHz using an Intan RHD2000 recording system. Offline, spikes were detected and automatically sorted using the Kilosort algorithm^37^ followed by manual curation using Klusters^38^. Analysis was performed in MATLAB using custom scripts. To measure the effect of laser light on spiking, peristimulus time histograms (PSTHs) were built around stimulus onset (spike trains were binned into 10-ms bins). Baseline and light-induced firing rate were calculated for each single unit. Baseline was defined as light-free epochs (1 s) between trials and stimulation period as the 405 and 635 nm light was on (300 ms). Wilcoxon-signed rank test was used to compare the mean firing rate per trial during baseline and laser stimulation.

## Results

### Optical design

**Fig. 3:**
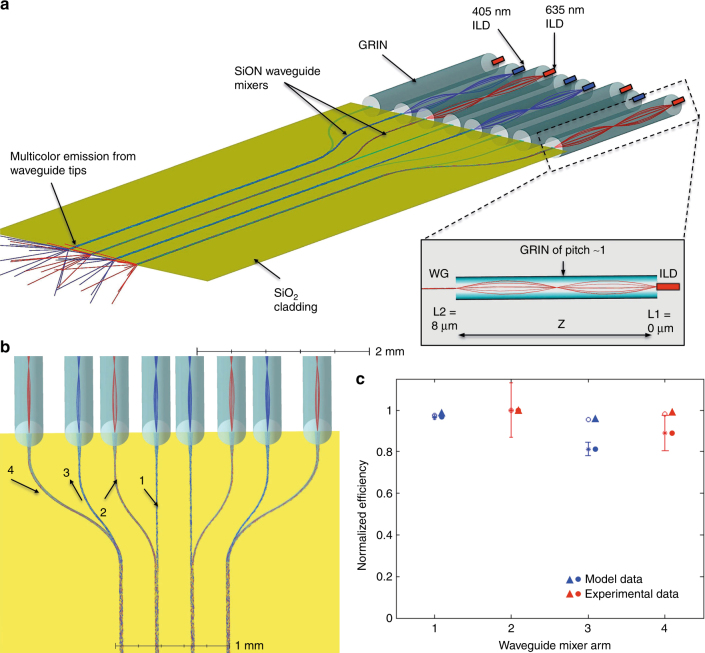
Optoelectrode optical design and light propagation. **a** Zemax optical model of optical mixer waveguide coupled to ILDs to deliver multicolor light output at all waveguide ports. The model consists of eight ILDs (four 405 four 635 nm ILDs) coupled to arms of optical mixer via their respective GRIN lenses. The 405 nm (2.38 mm long) and 635 nm (2.54 mm) GRIN lenses were designed and simulated in Zemax to facilitate optimal coupling while allowing maximum misalignment tolerance between the ILDs and the waveguide. The focused beam enters the waveguide mixer arms, which taper down from a width of 50 μm and converge into a 5 mm-long straight waveguide (cross-section: 30 μm × 7 μm). The schematic in the inset shows a full pitch GRIN lens collimating and focusing a divergent ILD laser beam into the waveguide mixer arm (WG). GRIN design parameters including N.A., working distances (L1 and L2) and mechanical length (Z), were optimized to achieve the desired magnification (*M* < 1) for enhanced optical coupling^27^. L1 and L2 denote object and image distances, respectively, that can fit well within the device fabrication and assembly precision. **b** Waveguide mixer arm geometries designed in Zemax to achieve optical output within ~3% of the mean value for both transmitting wavelengths (405 and 635 nm) at all waveguide ports. Mixer arm 1 is a straight waveguide. Mixer arms 2 and 3 are identical with 2.335 mm bending radius, but arm 2 transmits 635 nm wavelength and arm 3 transmits 405 nm wavelength, resulting in difference in optical intensities delivered at the output of each arm. Mixer arm 4 has the maximum bend of 1.370 mm radius. Since 405 nm wavelength suffers more dispersive loss than 635 nm, mixer arm with minimum loss (arm 1) was designed to transmit 405 nm and mixer arm with maximum loss (arm 4) was designed to transmit 635 nm. **c** Zemax simulated data and experimental data for normalized optical power emitted at the tip of each waveguide when transmitting at their respective wavelengths. The mixer arms 1 and 3 transmit 405 nm wavelength and are marked in blue; mixer arms 2 and 4 transmit 635 nm wavelength and are marked in red. Though mixer arms 2 and 3 have same geometric design, optical transmission of 635 nm wavelength via arm 2 is more than of 405 nm wavelength via arm 1 because 405 nm gets more scattered than 635 nm wavelength. The experimental optical output of all four shanks was within 11.4% of the mean value for both transmitting wavelengths. The measurement data was collected on optical micromanipulators (for *N* = 3 devices x 8 waveguides each) as described in Supplementary Information

The optical design was studied using ray-tracing models (Zemax LLC, Kirkland, WA, USA) and is shown in Fig. 3a, b. This design yielded an optical output at all shanks within 3% (Zemax model) and 11.4% (actual implementation) of the mean value for both wavelengths (Fig. 3c). The coupling interfaces at the ILD-GRIN and GRIN-waveguide junctions were modeled, yielding a total coupling loss of 0.925 dB from ILD to waveguide (coupling efficiency of 89.9%). The mixer arms were bent to route two colors to each of the four shanks. A multi-shank design involves the alignment of eight ILD-GRIN-waveguide pairs in all three axes, making it critical to keep designed losses to a minimum. The radiation losses in bends were reduced by maximizing the bend radius of mixer arms, which is limited by the diameter and assembly pitch of GRIN lenses. For maintaining the smallest possible form factor and maximizing the radius of curvature of each mixer arm, we designed mixer arm 2 (on shanks 2 and 3, red ILD) and 3 (on shanks 1 and 4, violet ILD) with bend radii of 2.335 mm; and mixer arm 4 (on shanks 1 and 4, red ILD) with bend radius of 1.370 mm^39,40^ (Fig. 3b). The width of all mixer arms was 15 μm, except arm 4 which had a width of 24 μm to compensate for the higher radiation loss in the sharper bend. Other than coupling and radiation loss, light rays also suffer from propagation loss, which is attenuation in the form of scattering and absorption as they travel through the guide. Since 405 nm suffers more scattering losses than 635 nm, ILDs were arranged in such manner such that 405 nm ILDs were coupled with mixer arms with the lowest loss (arm 1) and 635 nm ILDs were coupled with mixer arms with the highest loss (arm 4). Note that both colors are available on every shank. The idealized angle of light exiting the waveguide was calculated to be 18.11° (NA = *n*_0_* sin*θ*; *n*_0_ for brain tissue, 1.36; NA for the waveguide, 0.4228).

### Thermal design

Since temperature affects the mechanism of neuronal spike generation in various ways^41–43^, we have previously^27^ developed a bio-heat transfer model using COMSOL Multiphysics (COMSOL Inc.) to simulate temperature changes during optoelectrode operation. When applied to the present four-shank device, simulation results indicated that all 8 ILDs (two per shank) could be pulsed simultaneously (200 ms pulse width, 10% duty cycle) for 10 s continuously, which is more than adequate for many optogenetic circuit-analysis applications^4^ (see Supplementary figure, S1). By contrast, butt-coupled optoelecrodes (without an intermediate GRIN lens used for coupling) show a fast and oscillatory temperature rise at their probe shanks in response to the pulsed ILD driving currents indicating its limited practical use for multi-diode assemblies.

### Electrical design

#### EMI coupling in diode-based optoelectrodes

**Fig. 4:**
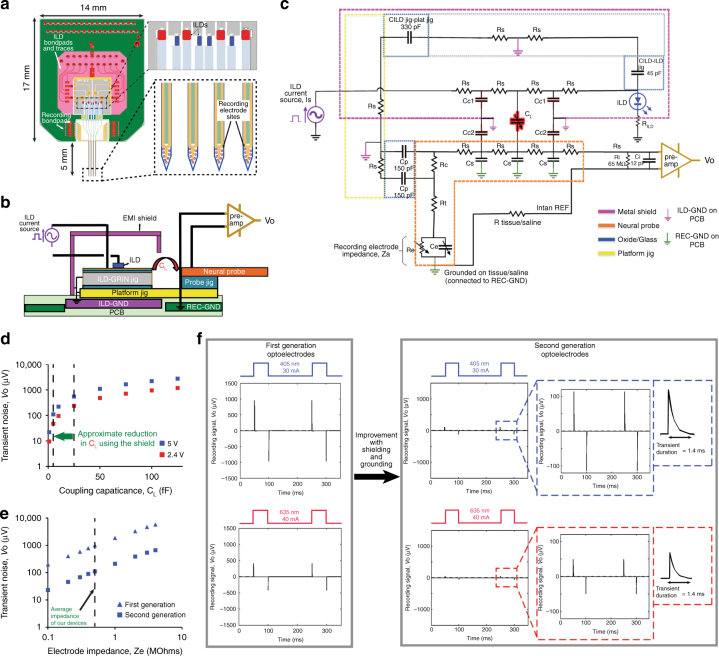
Lumped circuit model to study and minimize electro magnetic interference from stimulation traces to recording traces of the assembled optoelectrode. Front view of the assembled optoelectrode on a custom-designed PCB showing arrangement details and physical separation between ILDs, recording electrodes, ILD traces and recording traces. A representative block diagram of the optoelectrode assembly shown in **a** used as a reference to construct the lumped circuit model shown in **c**. **c** Lumped circuit model designed in Cadence SPICE simulator. The circuit blocks are color coded to the assembly components shown in **b**. The circuit was studied to minimize coupling noise from ILDs to the recording electrodes that gets picked up as high frequency transients at Intan output, *V*_O_. *C*_L_ is the stray capacitance coupling from ILDs to the neural probe due to the leaky metal shield around ILD-GRIN jig assmebly. Components *R*_S_ and *C*_S_ in the model are parasitic resistances and capacitances of silicon and the model were simulated for 1–10 Ω of *R*_S_ and 1–10 pF of *C*_S_. Ze is the impedance of electrode recording site on the neural probe and was assumed to be 500 kΩ unless Ze was the variable under study (Fig. 4e). The values of *R*_C_ (contact resistance of wire bonding pads of the probe) and Rt (transmission resistance of metal interconnects on the probe) were simulated for the range of 1–100 Ω. Ri and Ci (input resistance and capacitance of Intan differential amplifier) were modeled as 65 MΩ and 12 pF, respectively, based on the product specification sheet48. The probe was assumed to be grounded in saline or tissue; which ultimately connects to the recording ground of the PCB (PCB_REC-GND_). Other capacitances (C_ILD-ILD jig_; C_ILD jig-plat jig_ and C_P_) approximate parallel plate capacitances between different micro-fabricated silicon components. **d** The magnitude of stimulus-locked noise transients at V_O_, as a function of coupling capacitance, C_L_ (in absence and presence of a metal shield). The reduction in CL from ~30 fF to ~5 fF with the use of an EMI metal shield proved to be successful in reducing the transients at VO to less than 100 μV. The plot also shows dependence of noise transient magnitude on ILD stimulating voltage (~5 V for 405 nm and ~2.4 V for 635 nm). Electrode impedance, Ze, is assumed as 0.5 MΩ (Re = 1.12 MΩ, Ce = 284 pF). **e** The magnitude of stimulation-locked transients induced by 405 nm ILD as a function of electrode impedance, Ze for first-generation optoelectrodes^27^ (without grounding and shielding, assumed *C*_L_ = 30 fF) and second generation optoelectrodes presented in the current work (with shielding and grounding strategies implemented in the circuit model shown in (**c**), assumed *C*_L_ = 5 fF). The current model **c** above shows smaller increase in transient magnitude with increase in Ze, a desirable design characteristic for chronic studies. **f** Reduction in transient magnitude from first generation to second generation of optoelectrodes as predicted by the circuit model. The transients reduce from 1.55 mV (for 405 nm ILD) and 0.65 mV (for 635 nm ILD) in first generation optoelectrodes to 110 μV (for 405 nm ILD) and 50 μV (for 635 nm ILD) in second generation optoelectrodes (assumed *C*_L_ = 5 fF). The decay time of the transients remains the same for all cases when simulated for same values of electrode impedance (magnitude = 0.5 MΩ, phase = −65^o^)

In diode-associated neural probes, electromagnetic (EM) fields are generated by electric traces carrying modulating currents that drive the diodes. The abrupt rise and fall of modulating waveform charges and discharges the diode anode and cathode and this sudden change in EM fields, if picked up by the nearby high-impedance recording circuit, induces artifacts in neural data. In the present implementation (Fig. 4a), current-carrying ILDs are flip-chip bonded on the ILD-GRIN jig, and the entire surface of ILD-GRIN jig acts as a potential source of EM fields, which is coupled to the recording channels if not shielded. The fields may be complicated due to the presence of various coupling paths, which do not show up explicitly in a formal circuit. We therefore developed a lumped circuit model for analyzing electromagnetic interference (EMI) coupling paths in an opto-electrical assembly, and compared simulation results (SPICE, Cadence) of that model with measured (experimental) data. Lumped circuit analysis is a useful approach for predicting EMI behaviors^44–48^; especially at low frequencies, where most RF-targeted EMI analysis tools fail to converge to reliable results.

#### Equivalent circuit model

The assembly components (neural probe, probe jig, ILD-GRIN jig and platform jig) along with the ILD current source (EMI source) and recording channels (EMI victim) were modeled in SPICE circuit simulator (Fig. 4b). Initial analysis showed that coupling capacitance C_L_ from ILD to recording traces, even when as small as a few fF, dominates the magnitude of transients on the recording channel. In contrast, the effect of coupling inductance was negligible (modeled separately). Inductive effects began to be substantial at coupling distances on the order of μm, whereas in the present design distances were on the order of mm. These predictions were in good agreement with our bench tests, which showed a monotonic increase of the magnitude of the transients with d*V*/d*t* (the temporal derivative of the ILD driving voltage), but none with d*i*/d*t* (the temporal derivative of the ILD driving current). We thus focused our efforts on a lumped RC circuit model for studying the effect of different resistive and capacitive elements on the characteristics of stimulus-locked transients.

Figure 4c shows the lumped circuit (RC) model of our assembly design. Different model blocks correspond to their respective color coded assembly components shown in Fig. 4b. The model contains essential coupling paths between the ILD-GRIN jig and the neural probe that allow physical interpretations. We assume that the path of currents from the source (*I*_S_) to the ILDs is the primary noise source. The current source modeled as generating a 50 ms pulse of 30–50 mA. As current flows into ILD, the ILD turns on and a voltage (*V*_ILD_) is generated across its terminals. This voltage, when coupled to recording traces on the neural probe, generates a high frequency transient on the recording channel output, *V*_O_. *V*_ILD_ can be coupled to the recording electrodes via two paths: air and/or the solid conductors, e.g. the conductive silicon. The magnitude of capacitive coupling via air can be approximated by the equation, *C* = *πε*_*o*_*ε*_*r*_/ln(*d*/*r*) *F*/*m*^49^, which quantifies the parasitic coupling capacitance between parallel circular conductors (in this case, the ILD and the recording traces, plus their respective wirebonds) of an identical radius *r* separated by a distance *d*. *ε*_o_ and *ε*_*r*_ are the relative permittivity of vacuum and coupling medium (air) respectively. Using empirical values (*d* = 3 mm; *r* = 25 μm; *ε*_o_ = 8.85 × 10^−12^ *F*/*m*; *ε*_*r*_ = 1) the parasitic capacitance C_C_ was estimated to be ~30 fF. The crosstalk via the platform jig is governed by the size and thickness of all components that physically connect the ILD lines to recording channels. This was approximated by calculating individual capacitances between component pairs (ILD and ILD-GRIN jig, C_ILD-ILD jig_; ILD-GRIN jig and platform jig, C_ILD jig-plat jig_; platform jig and neural probe, C_P_) using the parallel plate capacitance equation: *C* = *ε*_o_*ε*_*r*_*A*/*d*; where the capacitance between two parallel surfaces depends on their overlapping area, *A*, and separation distance *d*. While a parallel plate capacitor underestimates the capacitance by ignoring fringing fields, later modeling (via increasing the calculated capacitances by several 10 s of pF) showed these values to have negligible effects. Shielding and grounding were previously shown to result in cancellation of diode-induced noise in manually assembled diode-probes^4^. We implemented design techniques in our micro-fabricated assembly to allow quick transient discharge to ground over shorter electrical paths. These techniques were first verified in the SPICE model by testing several discharge paths and shielding and grounding strategies. First, the floating silicon of ILD-GRIN jig and platform jig were both grounded (shown as PCB _ILD-GND_ in Fig. 4c). Second, a metal shield (pink in Fig. 4c) was added around the ILD-GRIN jig assembly, and grounded to PCB _ILD-GND_. However, the shield is imperfect since there would always be an opening at the front of the shield, required to enable coupling the GRIN lenses to the on-probe waveguides. Thus, the shield could not be modeled as perfect, and thus although most of the coupling capacitance (represented as *C*_C1_ and *C*_C2_ in Fig. 4c) could discharge to ground, a small fraction of escaping electric fields could still couple to the neural probe through the gap. This leak capacitance *C*_L_ was simulated for a wide range of values (1–125 fF), to represent the possibility that some assemblies may have higher *C*_L_ than others. All other circuit components including path resistances, coupling capacitances and parasitic components were estimated by calculations and making simplifying assumptions about the geometry of the components and tracks.

When the model was simulated with 30 mA (for 405 nm, 5 V) and 40 mA (for 635 nm, 2.4 V) driving currents to the ILDs, the stimulus-locked transients at *V*_O_ were found to be most sensitive to *C*_L_; a change of only a few femtofarads resulted in a logarithmic increase of the predicted magnitude of *V*_O_ transients (e.g., from 10 μV at 1 fF to 100 μV at 3 fF; Fig. 4d). Compared to 635 nm ILDs, 405 nm ILDs have a higher forward voltage (higher *V*_ILD_) and thus 405 nm ILDs induced higher magnitude transients at Vo (compare blue and red points at Fig. 4d).

Transient magnitude showed an increase with increase in electrode impedance, *Z*_e_. An increase in *R*_C_ also increased the transient magnitude but this effect was not evident until change in *R*_C_ was in the range of MΩ which is unlikely in practice. Figure 4e shows the transient magnitude vs. *Z*_e_, comparing two cases: with no shielding and floating silicon jigs (first generation design^27^); and with a low-resistance shield in place and assembly jigs grounded (second generation design, present work). In absence of a grounded shield, there is no path for *C*_L_ to discharge and hence all of *C*_L_ (~30 fF for ~3 mm of coupling length) is coupled from the ILD to the neural probe, giving rise to large transients (Fig. 4d). The floating silicon jigs also discharge to ground via their parasitic capacitances (Cp; not shown in Fig. 4d as Cp becomes redundant when the assembly is grounded), increasing the coupling capacitance. In other words, if shielding and grounding are removed from the model (all PCB_ILD-GND_ connections disconnected), the transients at *V*_O_ increase by a factor of ~10 and become more susceptible to the changes in geometry, sizes and parasitic electrical properties (resistance, *R*_S_ and capacitance, *C*_S_) of the assembled components and their physical separation. This case is similar to the first generation devices (which had only a ground plane but no shield and displayed in vivo transients in the mV range^27^). An un-grounded and un-shielded architecture also results in a higher change in noise over a given impedance range; an undesirable property since during long-term recordings electrode impedance may change over time.

Only grounding the jigs (assumed *C*_L_ = 30 fF) reduced the transient magnitude ~1.5 times, whereas only shielding the ILD-GRIN assembly (assumed *C*_L_ = 5 fF) reduced the magnitude ~2.4-fold. When both shielding and grounding were implemented together, transient magnitude was reduced ~10-fold (Fig. 4f). These simulations quantify how capacitive coupling via each path (air and solid conductor) produces substantial EMI at *V*_O_, giving relative sensitivity to the various design components and a solid rationale for the shielding and grounding approach implemented in the first diode-probes^28^. More widely, the present modeling approach, based on lumped circuit analysis, can be applied to a wide range of cases for understanding, diagnosing and approximating EMI behaviors in the low frequency (non-RF) regime.

#### PCB design

The optoelectonic array was constructed by assembling micro-fabricated components on a custom PCB and wirebonding them to the PCB bondpads. To minimize capacitive coupling between the light sources and recording traces, the PCB was designed with four planes (Fig. 4a, b). The top-middle plane (pink) is the ILD ground plane (PCB_ILD-GND_), over which the ILD traces are routed on PCB front side. The bottom–middle plane (green) is the recording ground plane (PCB_REC-GND_), over which recording traces are routed on the PCB backside. These two planes were designed so there was no overlap (in the *Z*-dimension) between the two planes to minimize capacitive coupling between them but it is unclear whether this is necessary.

### Optical characterization

**Fig. 5:**
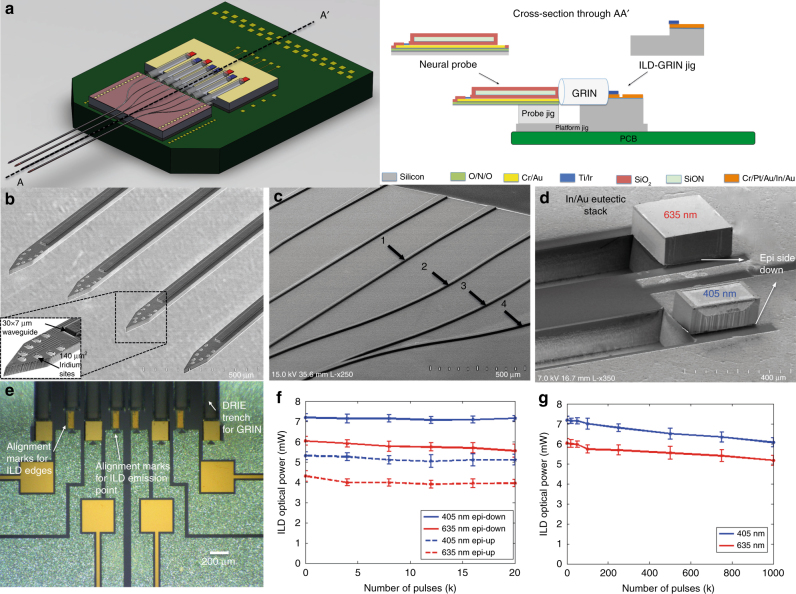
Fabricated components, ILD assembly and ILD lifetime testing. **a** Device fabrication details along A-A’ showing final assembly of fabricated components on PCB. The probe fabrication was carried out on a silicon-on-insulator (SOI) wafer with 22-μm thick device layer. The fabrication steps consisted of deposition of an LPCVD (low-pressure chemical vapor deposition) dielectric stack for stress compensation and electrical insulation; metal lift-off for interconnects, bondpads and electrode sites; deposition of PECVD (plasma-enhanced chemical vapor deposition)-grown waveguide (2-μm thick silicon dioxide (RI = 1.46) as bottom cladding, 7 μm thick silicon oxynitride (RI = 1.52) as core); patterning of dielectric mixer waveguides; deposition of another 2-μm thick silicon dioxide as upper cladding; contact opening for electrodes and bondpads; finally followed by probe shape definition and backside release using reactive-ion etch processes. ILD-GRIN jig fabrication started on a silicon wafer with 2-μm thick oxide for metal passivation. The process steps for the ILD-GRIN jig fabrication included lift-off of 6-μm thick indium stack to define ILD contacts and alignment marks to align ILD edges and ILD emission point; reactive-ion etching step of ~125 μm deep grooves as GRIN slots and a final dicing step to release ILD-GRIN jigs. Platform jig fabrication consisted of a single dry etch step to define shape followed by a backside release process. Probe jigs were released via dicing a wafer of a given thickness (no-mask process). **b** Fabricated neural probe shank tips with monolithically-integrated dielectric waveguides. The inset shows high magnification SEM image of a single shank with dielectric waveguide tip (7-μm core with 2-μm top and 2-μm bottom cladding) and iridium electrodes in Buzsaki8 configuration. **c** Fabricated dielectric waveguide mixer arms on the neural probe backend. All waveguide mixer arms (design 1, 2, 3, and 4) taper from 50-μm width at the backend to 30-μm width at shank tip. **d** High magnification SEM image of epi-side down flip-chipped 405 nm and 635 nm ILDs on the ILD-GRIN jig (heat sink made of silicon with 6 μm eutectic In/Au metal stack). **e** Fabricated ILD-GRIN jig (5 mm × 5 mm) with defined ILD alignment marks and eight bonded ILDs. **f**, **g** ILD characterization. **f** Comparison of optical power output and its decay for epi-side down and epi-side up flip-chipped ILDs (*N* = 5 for each ILD type, data points show the mean of the collected data and error bars represent standard deviation) when pulsed for 20,000 pulses at 5 Hz frequency, 20% duty cycle. Initial optical power of epi-side down bonded ILDs was measured to be 35.47% (for 405 nm at 30 mA) and 40.23 % (for 635 nm at 40 mA) more than that of epi-side up bonded ILDs. The optical power decay after 20,000 pulses was observed to be similar for epi-up and epi-down ILDs. **g** Lifetime testing of epi-down ILDs (*N* = 5 for each ILD type, data points show the mean of the collected data and error bars represent standard deviation) when pulsed for 1 million cycles at 5 Hz frequency, 20% duty cycle. The reduction in ILD output power after driving them through one million pulses was measured to be 18.94% for 405 nm ILDs and 16.12% for 635 nm ILDs when operated at 30 and 40 mA, respectively

The fabrication process (Fig. 5a) follows the Michigan probe microfabrication technology^31,50^ with addition of monolithically integrated dielectric waveguide mixers (Fig. 5b, c) and a custom silicon heat sink (ILD-GRIN jig) (Fig. 5d, e) for micro-optic assembly of ILDs and GRINs^27^. All device components were assembled on the PCB for optical device characterization.

#### ILD packaging and lifetime testing

Fluxless, no-pressure indium-gold eutectic bonding at 200 °C was used for bonding the ILDs to the ILD-GRIN jigs in an epi-side down configuration (anode facing down)^51,52^. This bonding recipe was selected to protect the ILDs from potential damage at high bonding temperatures and pressures. Indium is a soft solder that deforms plastically to form low stress eutectic bond with gold, it also offers high thermal conductivity (80 W/m/°C) and low electrical resistivity (88 nΩ-m). A bench-top laser diode driver (4201-DR, Arroyo Instruments) was used for characterization tests. The effectiveness of epi-side down bonded ILD technique was verified experimentally in this work by comparing the ILD performance characteristics for epi-side up and epi-side down bonding techniques (Fig. 5f). The initial optical power measured for epi-side down bonded diodes was 35.47% (405 nm) and 40.23 % (635 nm) higher than the epi-side up bonded diodes and this difference in optical power was maintained through 20,000-pulsed cycles (*N* = 5 diodes; Fig. 5f). Given the superior performance of the epi-side down packaging technique, it was used for assembling all devices. It is important to assess the lifetime of the ILDs before integrating them into implantable devices for chronic use. There can be various reasons for diode degradation over time including thermal damage due to absorption of laser light, recombination enhanced defect motion or facet degradation due to non-radiative recombination. We examined the longevity of our packaged ILDs by driving them at maximum currents for one million cycles (20% duty cycle, 40 ms pulse width). The initial (at the first pulse cycles) wall-plug efficiency (or radiant flux) for epi-side down packaged diodes was 5.1% (405 nm at 30 mA, *N* = 5) and 6.8% (635 nm at 40 mA, *N* = 5). The increase in wall-plug efficiencies as compared to the first generation devices^27^ may be attributed to the improved ILD packaging technique using a flip-chip bonder (Lambda Flipchip bonder, Finetech, Germany). A uniform ILD-to-substrate contact helps dissipate heat, yielding higher wall-plug efficiency. The decrease in ILD light power after running through one million pulses was 18.94% (405 nm, *N* = 5) and 16.12% (635 nm, *N* = 5; Fig. 5f, g). These results predict sustained optical output of the ILD-based probes even after extensive use, a requisite for chronic implantation.

#### System optical loss

We quantified optical losses in each part of the system separately: (1) coupling losses at the ILD-GRIN and GRIN-waveguide junctions; (2) radiation losses in the bends and corners of the optical mixer; and (3) propagation losses through the waveguide. The total optical loss, averaged over all mixer types in fully packaged devices was 13 ± 0.7 dB for 405 nm (5% coupling efficiency) and 10.88 ± 1.24 dB (8.2% efficiency; mean ± s.d., *N* = 12 ILDs of each color from 3 devices, 8 waveguides each). This translates to an average output irradiance of 1714 mW/mm^2^ (360 ± 116 μW output power; mean ± s.d.) for 405 nm and 2523 mW/mm^2^ (530 ± 49 μW output power; mean ± s.d.) for 635 nm at the waveguide tip and a calculated intensity of 8 mW/mm^2^ (for 405 nm) and 25 mW/mm^2^ (for 635 nm) at a distance of 200 μm from the tip, values well above the threshold for activating useful opsins such as ChR2, Arch3, and ChrimsonR. (~2 mW/mm^2^ for ChR2 at 405 nm^53,54^ and ~7 mW/mm^2^ for ChrimsonR at 635 nm^15^). The details of optical loss calculations are provided in the Supplementary section of this work.

### Electrical characterization

#### Impedance and noise bench testing

*Ex vivo* impedance and noise measurements were done in phosphate buffered saline (PBS, 0.1 M, Fisher Scientific) with a digital data acquisition system (RHD2164 amplifier board and RHD2000 Evaluation System; Intan Technologies) and benchtop laser drivers (4201-DR, Arroyo Instruments, LDC202C, Thorlabs) connected to ILDs on the devices. The average impedance of recording sites was 514 ± 107 kΩ with 65 ± 3° phase at 1 kHz (mean ± s.d., *N* = 5 devices with 154/160 sites; 6 sites were disconnected), which for a 14 × 10 μm electrode site is sufficiently low to record neural signals with high signal-to-noise ratio. The Johnson noise produced by circuit impedance of 514 kΩ at room temperature for lowpass of 7.5 kHz is 7.96 μV *V*_RMS_. The actual baseline noise picked up by the recording channels in absence of light stimulation was 9.55 μV *V*_RMS_, which is consistent with the calculated value.

**Fig. 6:**
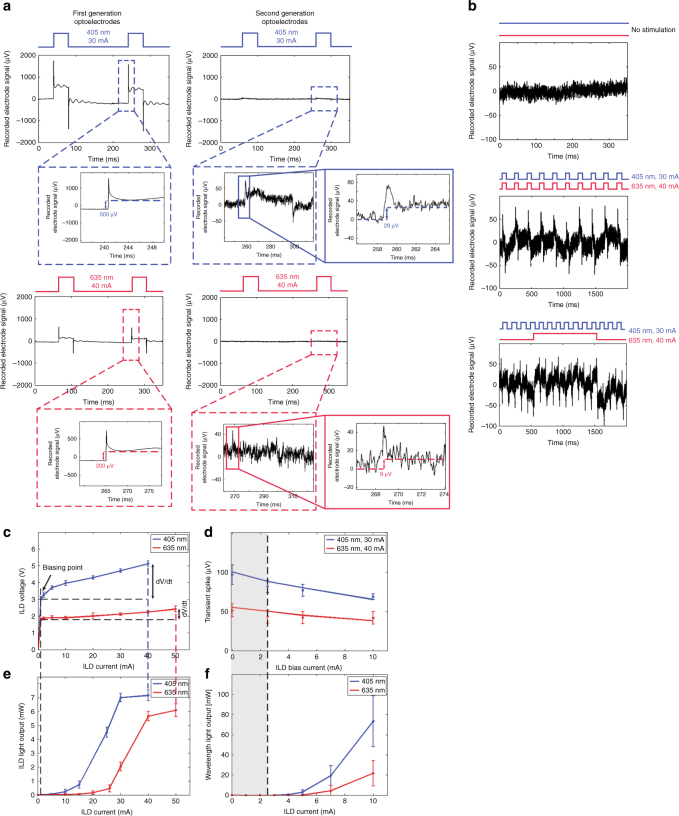
Ex vivo noise characterization. **a** Comparison of stimulation-locked artifacts between first-generation and second-generation optoelectrodes measured in phosphate buffered saline (PBS, 0.1 M) with an RHD2164 amplifier board connected to an RHD2000 Evaluation System (Intan technologies, Los Angeles, CA, USA). The transient magnitude and D.C. offset in the artifacts was reduced by a factor of ~15-24. 1.8 mV/ 0.5 mV mV (transient/DC offset) for 405 nm ILD (30 mA current) and 0.75 mV/ 0.2 mV (transient/DC offset) for 635 nm ILD (40 mA current) in first generation devices was reduced to 75 μV/ 29 μV (transient/DC offset) for 405 nm ILD (30 mA current) and 48 μV/ 11 μV (transient/DC offset) for 635 nm ILD (40 mA current). Due to the presence of the baseline noise and D.C. offset in the recordings, it is difficult to measure exact transient rise and fall times. **b** Baseline noise and stimulus-locked artifacts measured in phosphate buffered saline when Top, no ILDs are pulsed; middle, both ILDs (405 nm at 30 mA and 635 nm at 40 mA) are pulsed simultaneously at 40 ms pulse width and 20% duty cycle; bottom, 405 nm ILD is pulsed at 30 mA (20 ms pulse width, 20% duty cycle) and 635 nm ILD is pulsed at 40 mA (1 s pulse width, 50% duty cycle). **c** ILD voltage–current (V–I) characteristics for epi-side down flip-chipped 405 nm and 635 nm ILDs (*N* = 5; data points show the mean of the collected data and error bars represent standard deviation). When ILDs are biased at low currents (shown as ILD biasing point), the voltage differential (d*V*/d*t*) applied across ILD terminals decreases significantly. **d** Simulated and *ex vivo* measurements for stimulus-locked transient noise as a function of ILD biasing current. As the biasing current is increased, the voltage differential across the ILD drops, resulting in decrease in capacitive coupling and transient noise (*N* = 10 channels from the same device, data points show the mean of the collected data and error bars represent standard deviation). **e** Light output-current (L-I) characteristics for epi-side down flip-chipped 405 and 635 nm ILDs for the average range of ILD operation (*N* = 5; data points show the mean of the collected data and error bars represent standard deviation). **f** Waveguide optical power vs. ILD driving current for the assembled devices. No light output was detected at waveguide port for upto 3 mA of ILD current, confirming no possibility of neural stimulation at biasing, if implemented for ILD-GRIN optoelectrodes. (*N* = 2 devices with 8 waveguides each; data points show the mean of the collected data and error bars represent standard deviation)

Specifically, for the first generation 405 nm ILD (50 ms, 30 mA square pulse), the stimulation artifact amplitude was 1.8/0.5 mV (transient/DC offset); and for 635 nm ILD (50 ms, 40 mA square pulse), the amplitude was 0.75/0.2 mV (transient/DC offset) (Fig. 6a). There is also a turn-off transient which was proportional to the onset transient but lower in amplitude and of opposite polarity (Fig. 6a). The artifacts in second-generation devices measured only 75/29 μV (transient/DC offset) for 405 nm ILD (30 mA current) and 48/11 μV (transient/DC offset) for 635 nm ILD (40 mA current). The total artifact reduction (for transient and DC artifacts) was ~15–24 times. We also measured artifacts for different cases of simultaneous dual-color square pulse stimulation, which may occur during multi-opsin optogenetic experiments. Artifact magnitudes were maintained below 100 μV (Fig. 6b) for all cases with impedance values below 1 MΩ.

The artifacts observed in the bench testing comprised of fast turn-on and turn-off transients with asymptotic attenuation, similar to the capacitive effects predicted by our model (compare Fig. 4f with Fig. 6a). However, the experimental measurements also showed a DC offset component (Supplementary Figure S2). This offset was present when damaged ILDs (with same V–I characteristics but no light output) were driven too, suggesting that photoelectric-induced artifacts, if any, are minimal. This artifact also does not conform to any previous description of the photoelectrochemical artifact (or Becquerel effect)^29,55^. The photoelectrochemical effect is a function of light intensity and as previously reported, iridium in a buffered saline solution and induces a negative onset transient and positive turn-off transient. These artifacts were neither intensity dependent or of that polarity. The DC offset, a result of charge accumulation, could be due to a potential voltage difference between ILD ground and the recording ground level. The origin of this offset needs further investigation for future designs. Since lower impedance will further decrease the already low amplitude transient and DC offset (Supplementary Figure S2), we recommend any one of the many techniques currently available to lower the electrode impedance to future designers and users^56^.

#### Artifact reduction with ILD biasing

To further reduce EMI-induced artifacts in the system, we applied an ILD biasing technique, where a modulating current is superimposed on a DC current. We used very low currents (<3 mA), which correspond to high forward voltages for the ILDs (~70-80% of lasing threshold voltages; Fig. 6c), reducing the transient magnitude by 8–16% *ex vivo* (Fig. 6d). Biasing reduces the temporal derivative of the source coupling voltage, *V*_ILD_. Since the transients are due mainly to the capacitive coupling (*i* = CdV/dt), reducing the increase in voltage for a rectangular modulating waveform (dV/dt) reduces the coupling between the ILDs and the recording electrodes/connections. This was verified both via modeling and bench testing (Fig. 6d). Though laser biasing helps to reduce transient magnitude on recording channels, two critical points must be addressed before using biasing in optogenetic applications: light leak and tissue heating. First, no light should be emitted from the waveguide tip during biasing, to prevent sub-threshold opsin activation. Thus, ILDs should be biased far below the lasing threshold currents. For the ILD-GRIN probes, the ILD light power at 2.5 mA was ~5 μW (Fig. 6e). In this subthreshold regime, ILDs do not lase and behave as LEDs. Thus, light coupling via the GRIN lens is negligible (Fig. 6f). Note that the GRIN-based coupling mechanism provides a non-linear filter for the in-coupled light power. Thus, the biasing technique must be assessed for light leak for every system separately and may not be applicable when light sources (ILDs or LEDs) are coupled directly to fibers/waveguides without an intermediate lensing mechanism^4,57^. A second potential concern with biasing optoelectrodes is accelerated tissue heating. When biasing at a constant low current, the power developed (e.g., 2.5 mA x 1.8 V for 635 nm) causes heat to accumulate at the device, reducing the duration for safe operation in vivo. In our COMSOL heat model, the total device operation time for GRIN-based optoelectrodes was reduced from ~10 s (in unbiased mode; Supplementary Figure S1) to ~6 s (when biased at 2.5 mA). To summarize, diode biasing in the ILD-GRIN probes reduces the transient artifacts by about 10% without risking accidental opsin activation, at the expense of about 40% reduction in continuous operation time.

### Electrophysiological recordings

The fully packaged diode-probes, each complete with 8 ILDs, 4 dual-color waveguide ports and 32 recording sites, were used to record neural activity from dorsal CA1 of awake PV-Cre mice (*n* = 3; two head-fixed and one freely-moving animals). The mice were injected with a mixture of two AAVs, driving the expression of ChR2 under the CaMKII promoter (in pyramidal neurons, PYR) and ChrimsonR under the PV promoter (in PV-expressing interneurons, PV-INT).

**Fig. 7:**
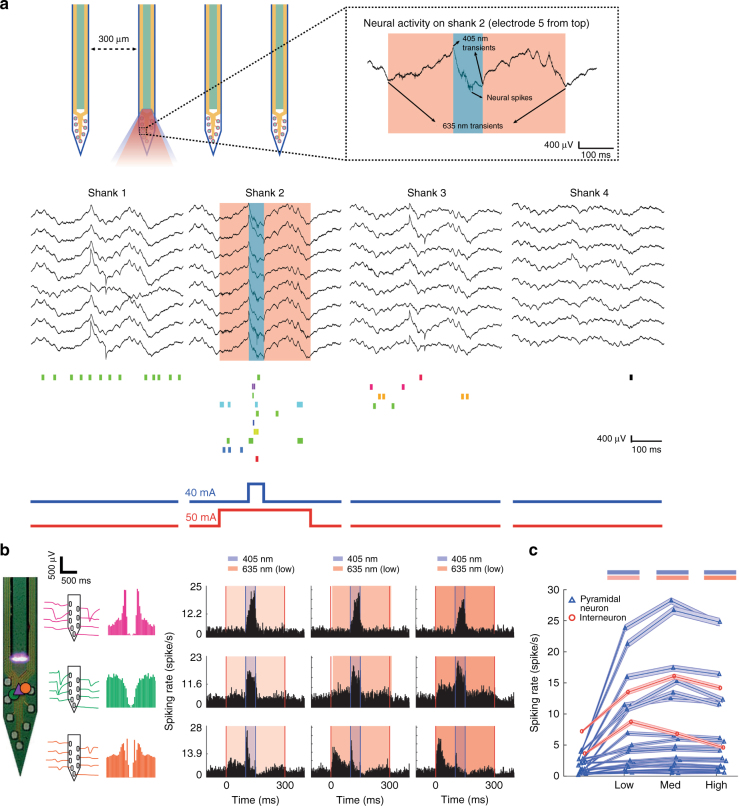
Control of multiple neural populations in vivo with ILD-GRIN waveguide probes. **a** Wide-band (0.1–7500 Hz) spiking activity recorded on a four-shank probe from CA1 pyramidal cell layer of an awake mouse expressing ChR2 in pyramidal cells and ChrimsonR in parvalbumin expressing interneurons. The illuminated shank shows spiking activity and light-induced artifacts during a 200 μW 405 nm light pulse (100 ms, 40 mA) and 450 μW 635 nm light pulse (300 ms, 50 mA). Note spontaneous activity on all shanks and induced spiking during ILD driving on illuminated shank. When simulated in our thermal model, the device can be driven for up to ~27 continuous seconds when driving 2 ILDs on the same shank with a total input electrical power of 320 mW (40 mA x 5 V for 635 nm + 50 mA x 2.4 V for 405 nm). **b** Independent dual color excitation of pyramidal neurons (PYR) and interneurons (PV). The spiking data was quantified for 37 well-isolated cells (35 PYR and 2 PV interneurons) recorded simultaneously from CA1 (same animal and session as in Fig. 7a). Inset of the probe tip shows the vertical location of three light-modulated cells (1 PYR and 2 PV) relative to the probe sites. Plots in the center show auto-correlation histogram and spike waveform (mean and SD) in the lack of any illumination. Histogram plots on the right show examples of spiking response to 50 ms long 405 pulses and 400 ms long 635 nm pulses (for three different intensities) in a ChR2 + PYR, and two ChrimsonR + PV. Note the light-modulated increase in spiking response of PYR cells and PV cells with 405 and 635 nm light, respectively. Also note the decrease in spiking response of a putative PYR (not shown), possibly making a synaptic connection to the orange PV cell, with higher intensity of 635 nm illuminations. More experiments and analysis are ongoing to study these effects and similar circuit effects observed in other cells. **c** Dual color modulation of single units (mean firing rate ± SEM are shown). Firing rate of 19 PYR (blue triangles) and 2 INT (red circles) were modulated by 405 nm (50 ms, 40 mA) and 635 nm light pulses (300 ms, 25, 30, and 40 mA)

Spontaneous neural activity was recorded on all shanks while light-induced neuronal activity was observed on illuminated neural shanks (Fig. 7a). As expected, illumination with 405 nm light pulses (100 ms; 40 mA, 200 μW) elicited spiking in 19/35 (54%) PYR and in 2/2 (100%) PV cells. 635 nm light pulses (300 ms; low/medium/high power: 45/50/55 mA, 340/420/500 μW) elicited spiking in a subset of cells (2/2 putative PV-INT, 100%) recorded on the same shank (Fig. 7b, c). An example PYR responding to 405 nm light (mean firing rate is 2.9 Hz without and 24.2 Hz with stimulation, *p* < 0.001; Wilcoxon signed rank test) and having no response to 635 nm light is shown in Fig. 7b (pink cell, *p* > 0.05; Wilcoxon signed rank test). The two putative PV-INT (Fig. 7b, green and orange) showed monotonically increasing spiking with increased 635 nm light power (6.06 Hz baseline firing rate increased to 6.16, 6.33, and 6.47 Hz for middle row and 5.57 Hz baseline firing rate increased to 11.17, 15, and 16.75 Hz for bottom row; *p* < 0.05 for both neurons in all conditions; Wilcoxon signed rank test), presumed to result from direct ChrimsonR-mediated depolarization. These same two cells also increased their spiking during 405 nm illumination (mean firing rates are 16.91, 15.3, and 12.97 Hz for middle row and 9.1, 6.5, and 4.4 Hz for bottom row at low, medium, and high conditions), presumed to result from synaptic inputs from the population of ChR2 expressing pyramidal neurons, which are known to make excitatory synapses on these neurons (Fig. 7b).

As expected the current results in Fig. 7 show effective light-mediated modulation of ChrimsonR with various levels of red light output, ~300–500 μW range, indicating responsiveness of ChrimsonR to red light. The observed light-modulated increase in spiking activity was also localized through the length of illuminating shank (Supplementary Figure S3), highlighting two characteristics of this approach. First, the waveguide light emission cone is narrow enough for the shanks to have statistically insignificant light-crosstalk between them. Second, the light intensity as low as 70 μW (for 405 nm) at the waveguide output is high enough to illuminate up to 200 μm of tissue depth. Results from our previous study^27^ had indicated that red light power of about 400 μW (range, 50–500 μW) was only partially effective at silencing spiking of nearby eArch3 neurons. Given the observation that optical silencers require higher light intensity than ChR2^1,21,22^, our current preparation was designed to have ChrimsonR, a red-shifted channelrhodopsin activator with high sensitivity.

For square pulse illumination, we recorded 120 μV transients for 200 μW violet-light 40 mA/5 V (50 ms), <50 μV transients for 450 μW red light illumination (50 mA/2.4 V/300 ms). In contrast, half-sine stimulation (peak power/current: 200 μW/40 mA) with 405 nm light yielded transient-free recording (Supplementary Figure S4). The electrical design modification discussed in this work was a useful modification since in vivo electrical artifacts were considerably reduced in these probes compared to the first generation^27^ though both were capable of robustly driving neural activity (Supplementary Figure S5). The ILD biasing technique for artifact reduction was also verified in vivo where the artifacts induced by 405 nm ILD (at 40 mA) were reduced from 120 μV at zero current bias to 97 μV when biased at 2.5 mA current. The magnitude and shape of the transient artifacts only minimally affected the spike sorting process, which resulted in well-isolated single units (Supplementary Figure S4). In this figure, the trial-to-trial variance of both spike activity and artifacts are plotted.

## Discussion

Our compact opto-electronic packaging (22 mm × 14 mm device size for 32 channel, 8 ILD-GRIN pairs, 1.5 g device weight) successfully demonstrates high fidelity electrical and optical signals, adequate light irradiance, heat removal and environmental protection for longevity, and re-usability. One of the biggest ILD micro-packaging challenges is to manage thermal dissipation, especially for applications that require driving lasers at wider-pulse widths (milliseconds are used to drive most opsins). Epi-side down bonding technique prevents this damage by bringing the heat source to the heat sink as close as possible and minimizing the thermal resistance^58,59^. The use of eutectic metals with good thermal conductivity and formation of void-free thermal contact between the laser diode and the heat sink further minimizes thermal resistance. A solder bump technique^58^ can accomplish chip positioning through self-alignment; however, thermal dissipation is compromised since heat can only be transferred through the solder bumps. Bridged die bonding^60^ employs solder pattern with a gap; it facilitates better heat conduction than solder bump technique but still provides a higher thermal resistance for the heat flux generated at the active region that has to be re-directed to the side of the laser diode before traveling towards the heat sink. Understanding these design considerations and maximizing heat management for our design, we designed the metal pads on our heat sink to have a full contact with the ILD anode face. The ILDs were positioned such that the front of their emitting ridge hangs over the GRIN trench by 10 μm, preventing the solder from blocking the front ridge. The implemented bonding technique used was a pressure-free bonding to ensure that the ridge does not sink in the solder. The diode bonding metal pads were designed to cover maximum area of the ILD-GRIN jig for facilitating maximum conduction and uniform heat distribution on the 5 mm × 5 mm heat sink. The shorter heat paths from ILDs’ active region to ILD-GRIN jig and finally to the ground plane of the PCB were modeled to verify safe and reliable operation for optoegentics experiments^27^.

We used a lumped circuit modeling approach for understanding the sensitivity of the physical and non-contact coupling paths in our device. The model simulations were successfully able to identify and mitigate the putative source of stimulus-locked artifacts associated with our first-generation ILD-GRIN waveguide probes. The grounded silicon jigs along the metal shield formed effective 6-face faraday enclosure for the electrical assembly, suppressing most of the emitting electric fields generated within the ILD-GRIN jig assembly. The grounded jigs made of highly conductive silicon wafers (0.005 Ω-cm resistivity) provide a physical path for the transients arising from the stimulation EMI sources to quickly discharge to the ground and the brass shield helps to terminate and ground the electric fields that couple via air. Other than providing electrical shielding, the shield also helped to block the light escaping from the optical coupling junctions and facilitate convective cooling during device operation via 200 μm diameter air holes drilled on its top surface. Air holes in the cap were much smaller than the working EM wavelengths and therefore provide almost no impedance to the flow of currents on the conducting cap surface and hence do not affect the shielding quality.

While facilitating thermal protection of ILDs and adequate electrical noise shielding at device backend, our effective diode packaging solution also enabled precise assembly of optical components with wide alignment tolerance. We were able to achieve high optical efficiency range of 4.2–10.86% for the assembled working prototypes (the highest reported efficiency for optoelectrodes with integrated light sources to date), more than sufficient for optogenetic studies. However, if an increase in coupling efficiency is required for some application, the GRIN lenses can be modified to have anti-reflective coating on their coupling faces, increasing coupling efficiency by ~7% (per manufacturer specifications) at an increased cost/lens. Another effective way of increasing overall system efficiency is to fine tune the waveguide film quality during deposition process, lowering propagation losses. 405 and 635 nm wavelengths were chosen for our current multi-opsin optogenetic study to have minimum overlap between their excitation spectrums; the laser diodes are also available at a size and cost that allows the system to scale. Using optical modeling in Zemax and experimental optical bench testing, we have previously investigated the effect of misalignment tolerances on optical efficiency of the GRIN-based optoelectrode^27^. The wide misalignment tolerance range offered by the GRIN lens helps maintaining reproducible device yield in a mass production.

## Conclusion: future direction and novel applications

There are several possibilities to expand the current dual color waveguide probe design for circuit control applications, starting with the increase in the density of stimulation and recording sites per shank or increase in number of shanks. With an ever-growing photonics industry, as bare laser chips become available in more wavelengths in near future, our technology can be readily extended to conduct multi-opsin experiments. Multiple-shank probes with multi-optical/electrode sites can be fabricated using the same fabrication process flow. For more compact designs, the waveguide widths can be reduced to route more than one waveguide on the same shank with multiple emission points per shank, and this has already been demonstrated^61^. Waveguides could also be patterned over the interconnection lines to optimize shank space. Nanophotonics are also continually developing novel coupling schemes for higher density waveguide arrays^62,63^. Integrating high-density designs with higher power ILDs and/or enhancing the waveguide film characteristics can compensate for the propagation loss through thinner waveguides. Interconnection width and pitch can be decreased using deep UV and electron-beam lithography techniques. These modifications will allow for narrower shank widths with more waveguide ports for every set of recording sites; and also provide sufficient waveguide light power for cell activation and/or silencing. The geometry and surface of waveguide apertures can be modified to create various light diffusion profiles for specific target applications. Two groups have demonstrated that conventional gratings can out-couple light with very little divergence^61,64^, which may have applications if one is seeking to illuminate deeper tissue outside the recording region or seeking to image at specific points. Alternatively, light diffusion could be made even more divergent than the approach presented here which would provide local illumination similar to micro-LED methods. Packaging modifications can also include integration of on-board ILD driving or wireless circuit control. Advanced EMI analysis tools (such as high frequency structural simulator) can also be studied for more extensive noise analysis in future designs.

This multicolor optoelectrode technology offers a compact design solution that can effectively stimulate and record neural spikes with less than 100 μV stimulation-locked transients on the recording channels while maintaining the optical and thermal design merits of an implantable neural optoelectrode. Our principal in vivo result is intensity modulated optical excitation of pyramidal cells and PV interneurons in the same volume of densely populated CA1 of hippocampus of awake mice. This technology specifically enables multiple cell type manipulation for the study of neural circuits and is potentially useful for protocols such as closed-loop bi-directional control and creation of creating unique spatiotemporal spiking patterns. By using precise multicolor light delivery, our technology can aid in understanding of the organization and function of complex neural circuits, an important and fascinating avenue of neuroscience research.

## Electronic supplementary material

Supplementary Movie S1

Supplementary Information(DOCX 3106 kb)
