# Supplementary material for: Dual color optogenetic control of neural populations using low-noise, multishank optoelectrodes
Source: Microsyst Nanoeng. 2018 Jun 4;4:10. doi: 10.1038/s41378-018-0009-2 (PMC6220186; doi:10.1038/s41378-018-0009-2)
Supplement: Supplementary file 2 — Supplementary Information(DOCX 3106 kb) [file 41378_2018_9_MOESM2_ESM.docx]

**SUPPLEMENTARY INFORMATION**

**Dual color optogenetic control of neural populations using low-noise, multishank optoelectrodes**

Komal Kampasi^1,6^, Daniel F. English^3^, John Seymour^2^, Eran Stark^4,5^, Sam McKenzie^3^, Mihály Vöröslakos^2^, György Buzsáki^3^, Kensall D. Wise^1,2^, *Euisik Yoon^1,2^

^1^Department of Biomedical Engineering, University of Michigan, Ann Arbor, MI 48105, USA

^2^Department of Electrical Engineering and Computer Science, University of Michigan, Ann Arbor, MI 48105, USA

^3^NYU Neuroscience Institute, School of Medicine, East River Science Park, Alexandria Center, 450 East 29th St, 9th Floor, New York, NY 10016, USA

^4^Department of Physiology and Pharmacology, Sackler Faculty of Medicine, Tel Aviv University, 69978 Tel Aviv, Israel

^5^Sagol School of Neuroscience, Tel Aviv University, 69978 Tel Aviv, Israel

^6^Center for Micro and Nanotechnology, Lawrence Livermore National Laboratory, Livermore, CA 94550, USA

(Lawrence Livermore National Laboratory is operated by Lawrence Livermore National Security, LLC, for the U.S. Department of Energy, National Nuclear Security Administration under Contract DE-AC52-07NA27344.

IM # LLNL-JRNL-747557)

**Supplementary Figure S1: COMSOL heat transfer model results for high-density optoelectrodes.**

Tissue temperature rise over time for multi-shank GRIN-coupled optoelectrodes compared to their design equivalent butt-coupled optoelectrodes with 8 and 16 assembled diodes. The power values on each graph line signify the total input electrical power delivered to the device, 80 mW per diode. Butt-coupled optoelecrodes show a fast and oscillatory temperature rise at their probe shanks in response to the pulsed ILD driving currents. In contrast, GRIN-coupled optoelectrodes exhibit slow and gradual temperature rise because of thermal isolation between ILDs and probe shank, offered by the thermally insulating GRIN lenses. For a conservative estimate, we assumed that all electrical input power of ILDs is converted into heat and loosely defined the design threshold as 1 °C temperature rise from the baseline tissue temperature of 37 °C^30,32^.

GRIN-based optical design offers high optical coupling efficiency; hence, optical power requirement for neural stimulation can be met by integrating low optical power ILDs. The use of low-power ILDs and thermally insulating GRIN lenses in our design minimizes excessive tissue heating during operation. This has a critical influence on the thermal budget when scaling up the device in terms of the numbers of shanks and diodes. As the number of diodes per device increases, the electrical power consumed, and hence the dissipated heat, increases.

**System optical loss calculations**

Measurement using the direct cut-back method was used to evaluate propagation loss per unit length of a straight waveguide^26^. The observed slope of the linear fit 0.48 dB/mm for 635 nm and 0.59 dB/mm for 405 nm, gives the waveguide propagation loss. The y intercept (at 0 mm length) of the linear fit, 1.76 dB for 635 nm and 1.92 dB for 405 nm, gives the total coupling (including Fresnel) loss between the GRIN lens and waveguide, including back reflection at the tip of the waveguide. The coupling loss from ILD to GRIN output was separately estimated as 0.5±0.1 dB for 635 nm and 0.6±0.05 for 405 nm (mean ± s.d., N=5) by comparing optical power at ILD and ILD-GRIN outputs. Radiation losses from straight channel waveguides are generally negligible for well-confined modes but may increase in waveguide bends. Our mixer geometry has two bends per light path, and we measured radiation losses of 0.93±0.47 dB for mixer arm 2 and 1.1±0.31 dB for mixer arm 4 (mean ± s.d., N=5) when coupled to 635 nm ILD source. Similarly, we measured radiation loss of 0.95±0.12 dB for mixer arm 3 (mean ± s.d., N=5) when coupled to 405 nm ILD source. The summed losses for all mixer arms during bench testing were 7.28±0.05 dB for arm 1 (405 nm source, 7.68 mm waveguide length), 6.9±0.57 dB for arm 2 (635 nm source, 7.728 mm waveguide length), 8±0.17 dB for arm 3 (405 nm source, 7.728 mm waveguide length) and 7.41±0.41 dB for arm 4 (635 nm source, 7.887 mm waveguide length) (mean ± s.d., N=5).

However, the optical loss measured for packaged devices (**Figure 2**) was 13±0.7 dB and 10.88±1.24 dB (mean ± s.d., N=3) for 405 nm and 635 nm, respectively. This is mainly due to increased misalignment errors in the micro assembly when aligning optical components on a common substrate (PCB) as compared to characterization done on micromanipulators.

**Supplementary Figure S2: Transient artifacts, DC offset artifacts and impedance recorded with ILD-GRIN optoelectrodes ex *vivo.***

***
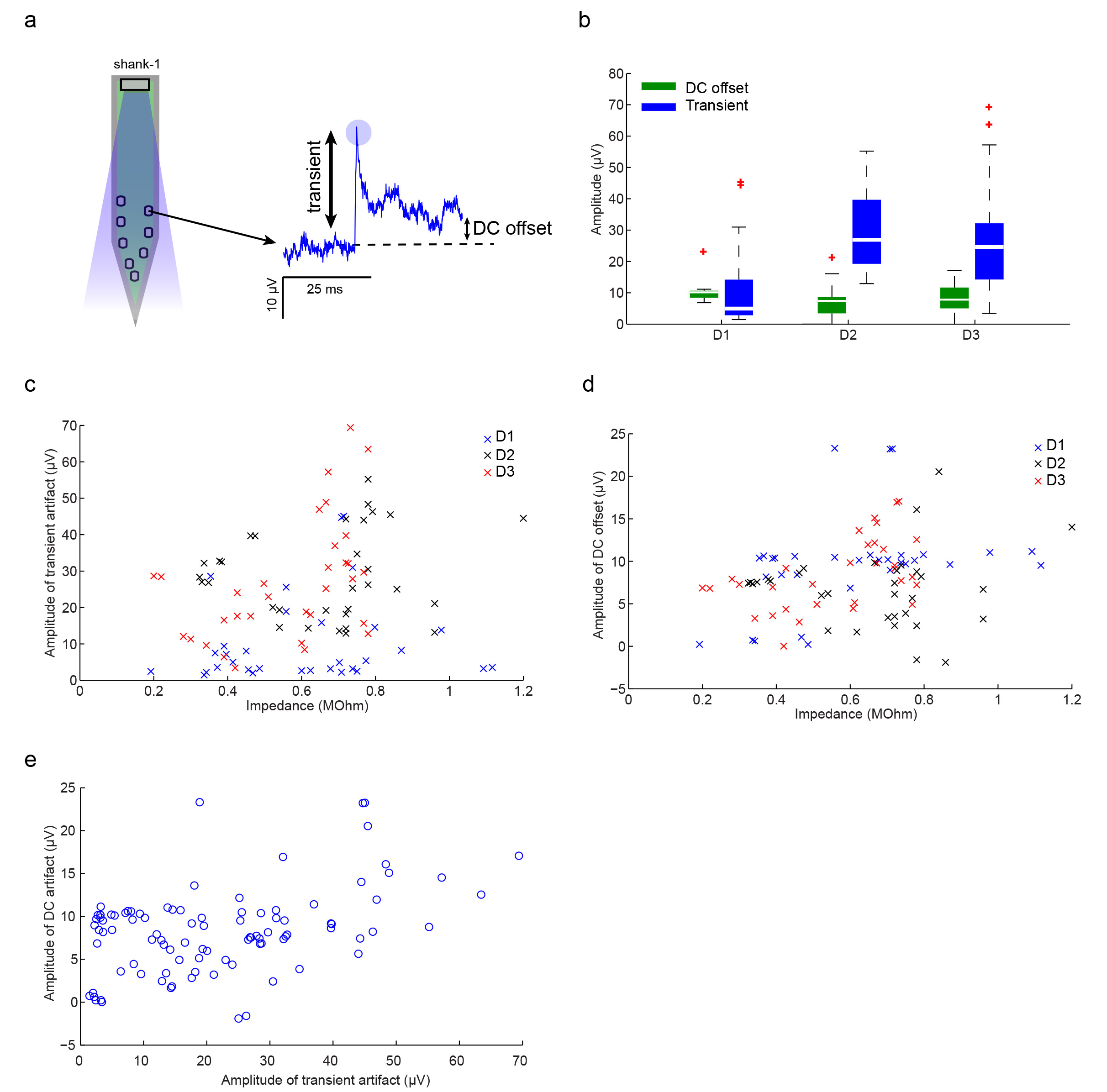
***

Transient and DC offset noise recorded ex-vivo (in saline) during 405nm stimulation. **(a)** Average waveform of 50 trials (blue ILD on, 40mA, pulse duration: 50ms). **(b)** Artifacts recorded from three four-shank devices (D1, D2 and D3). Transient artifacts are 10.46±12.04 µV, 29.03±12.18 µV and 26.56±16.48 µV for D1, D2 and D3, respectively; DC offset artifacts are 9.68±5.66 µV, 6.75±4.57 µV and 8.51±4.19 µV for D1, D2 and D3, respectively (mean ± SD values are reported). (**c, d**) Transient and DC artifacts plotted from three four-shank devices (D1, D2 and D3) as a function of electrode impedance. The plots show a correlation between the artifacts and the impedance of the respective electrode sites (r_s_=0.23 and p=0.02 for transient and r_s_=0.26 and p=0.01 for DC offset; Spearman correlation). (**e**) Correlation between transient and DC artifact on the same channel (r_s_=0.32 and p=0.0017; Spearman correlation).

**Supplementary Figure S3: Mean firing rate gain (color axis) as a function of shank number (x-axis) and vertical distance (y-axis) with ILD-GRIN optoelectrodes *in vivo*.**

Mean firing rate gain as a function of shank number and vertical distance (waveguide site at 0 μm) when shank 2 is illuminating 405 nm light while other shanks have no light on them. Each sub plot if for a different power level of 405 nm at the waveguide tip. The plots show the capability of ILD-GRIN probes to illuminate tissue depths of up to ~200 μms. The gain in firing rate seen at the bottom of shank 3 could be because of synaptic connections between neurons recorded on shank 2 and shank 3 or possible light crosstalk between the shanks.

**Supplementary Figure S4: Comparison of stimulus-locked transient artifacts and spiking activity of neurons in vivo.**

**
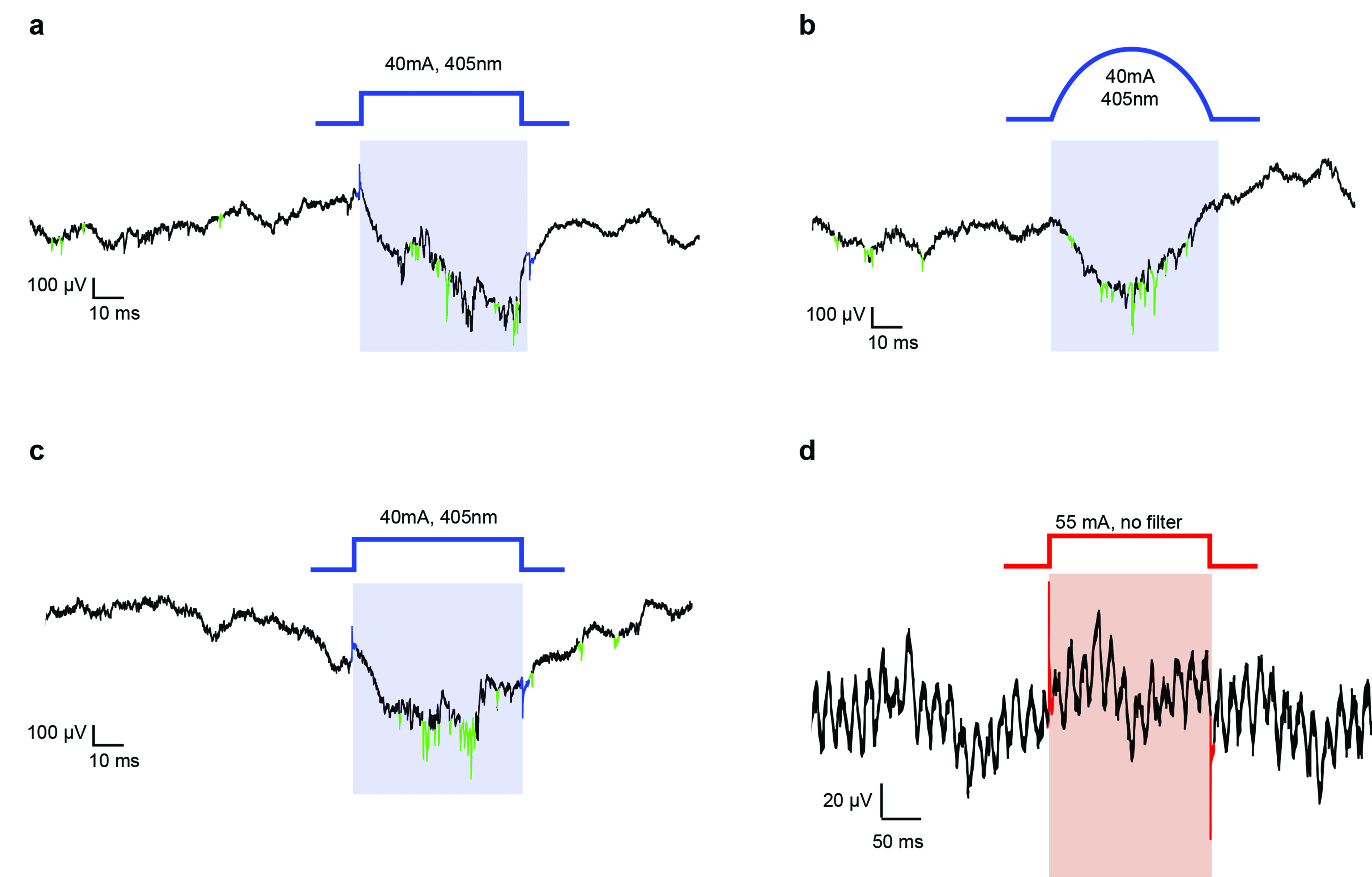
**

**
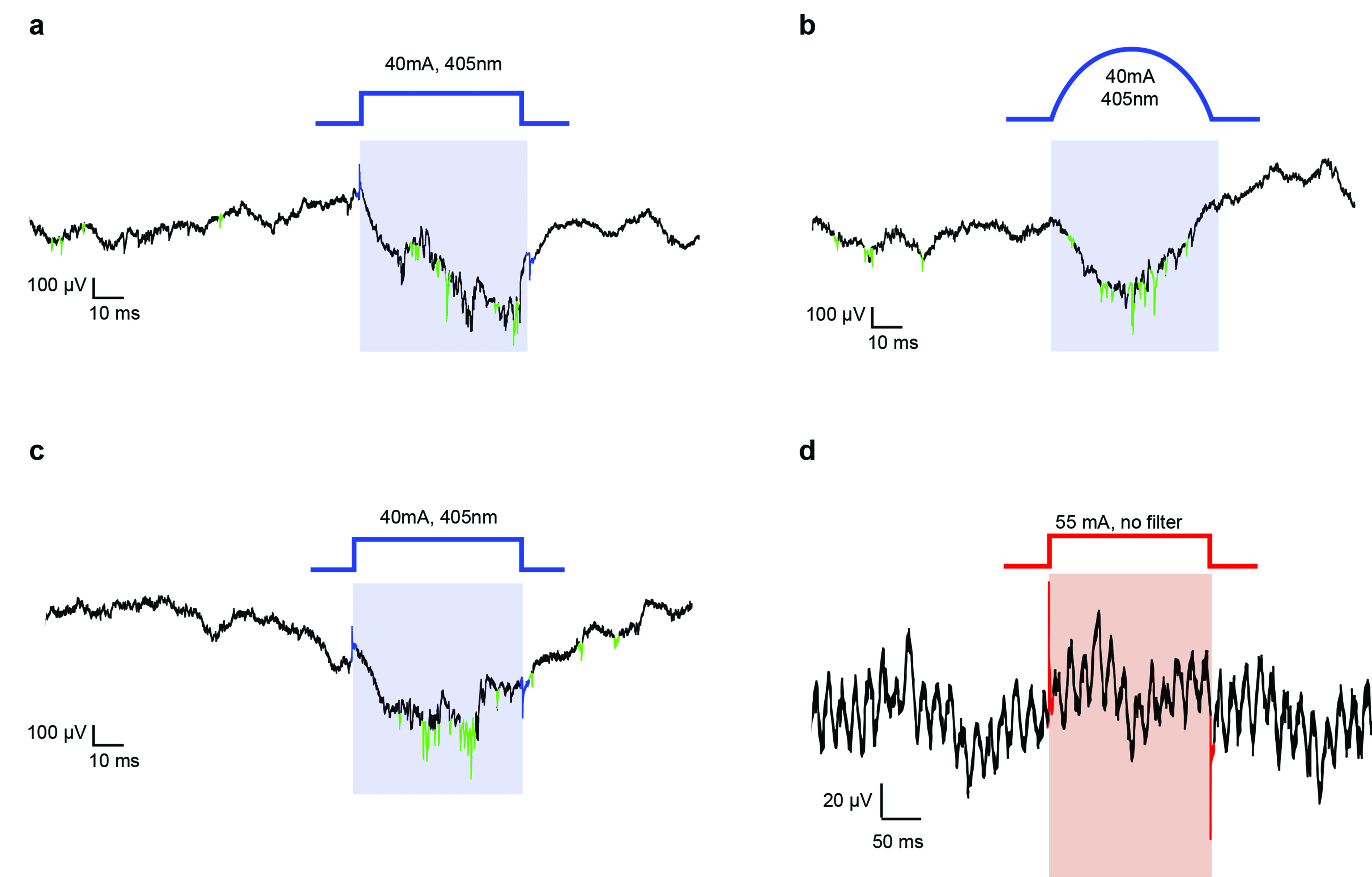
**

**
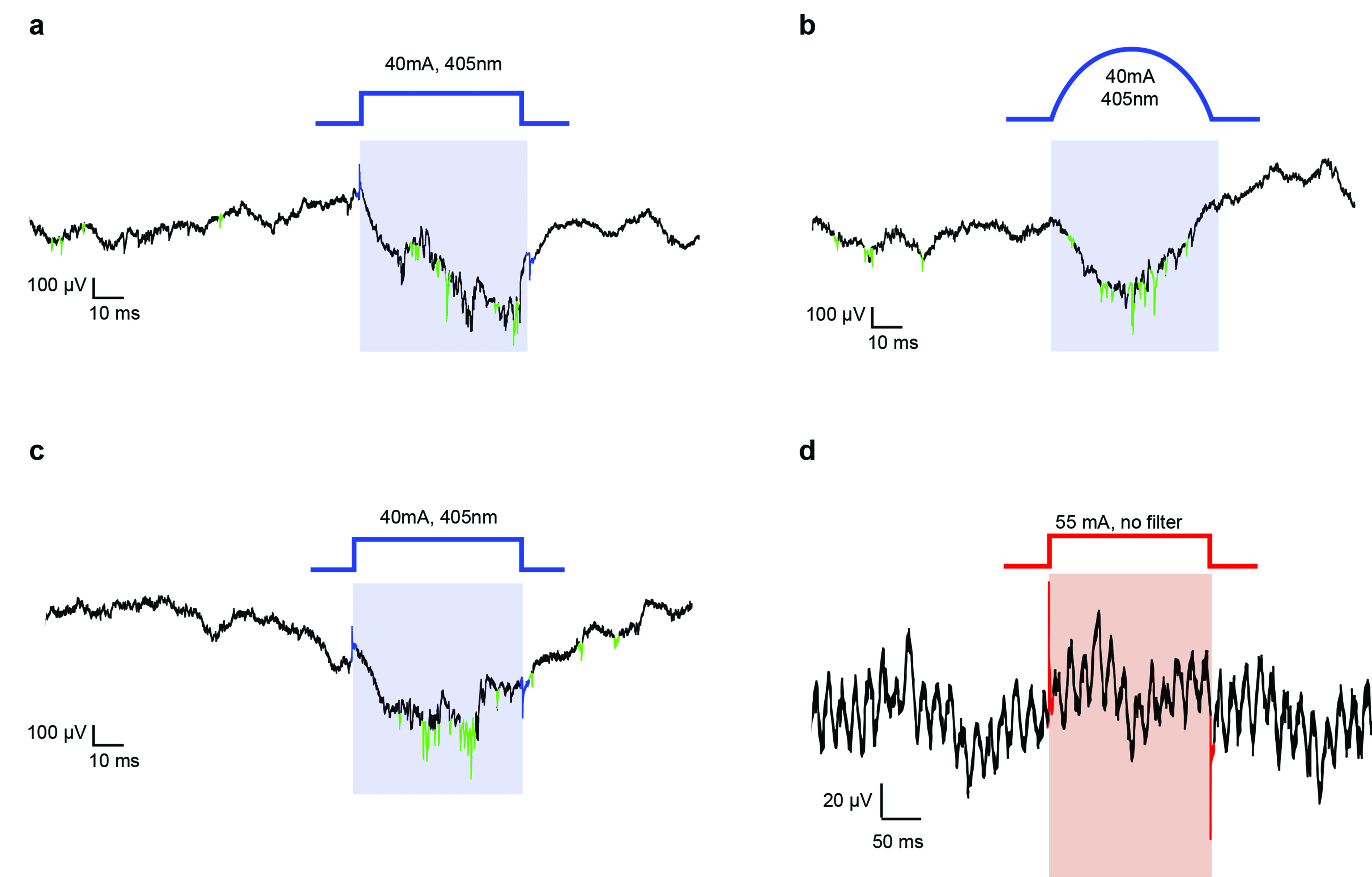
**

Wide-band (0.3-7,500 Hz) spiking activity and stimulus-locked artifacts recorded from CA1 pyramidal cell layer of an awake head-fixed mouse expressing ChR2 in pyramidal cells and ChrimsonR in parvalbumin expressing cells (interneurons)**. (a)** Measured 120 μV / -95 μV (onset/offset) artifacts for a 40 mA square pulse (405 nm diodes) and artifact-free recording for 40 mA half-sine stimulation (405 nm diodes) and 48 μV /-50 μV artifacts for 50 mA square pulse stimulation (with 635 nm diodes). Green traces highlight neuronal spikes shown on the same time-amplitude plot.

**Supplementary Figure S5: Comparison of stimulus-locked artifacts *in vivo* between first generation and second generation of ILD-GRIN optoelectrodes**

Qualitative comparison between stimulus-locked artifacts in first generation and second generation of optoelectrodes. The left panel shows the wideband spiking activity (0.3-10,000 Hz) recorded using first generation devices^30^; which measured 5 mV/ 2 mV/ -1.8 mV (onset/DC/offset) for 25 mA current (405 nm diode) and 3 mV/ 1.5 mV/-0.7 mV (onset/DC/offset) for 40 mA current (635 nm diode). With implementation of improved electrical design in current second generation devices, the recorded artifacts during light stimulation (as shown in the right panel) were significantly reduced to 120 μV/ -95 μV (onset/offset) for 40 mA current (405 nm diode) and 48 μV /-50 μV (onset/offset) for 50 mA current (635 nm diode).

**Supplementary Movie S1: Independent control of dual color light patterns generated at the output waveguide ports (30 μm x 7 μm)** **of a 4-shank ILD-GRIN coupled optoelectrode (in attachments).**

The video demonstrates intensity-controlled, independent activation of different wavelengths for an ILD-GRIN-coupled optoelectrode. The video begins with independent activation of 405 nm wavelength, then shows independent activation of 635 nm wavelength and finally switches to simultaneous emission of both 405 nm and 635 nm wavelengths at different output ports.
